# Quadratus lumborum block versus transversus abdominis plane block for postoperative analgesia in patients undergoing abdominal surgeries: a systematic review and meta-analysis of randomized controlled trials

**DOI:** 10.1186/s12871-020-00967-2

**Published:** 2020-03-02

**Authors:** Xiancun Liu, Tingting Song, Xuejiao Chen, Jingjing Zhang, Conghui Shan, Liangying Chang, Haiyang Xu

**Affiliations:** 1 Department of Anesthesiology, The First Hospital of Jilin University, No.71 Xinmin street, Changchun, Jilin 130021 China; 2 Department of Anesthesiology, China-Japan Friendship Hospital, Beijing, 100029 China

**Keywords:** Pain scores, Abdominal surgery, Quadratus lumborum (QL) block, Transversus abdominis plane (TAP) block, Meta-analysis

## Abstract

**Background:**

Abdominal surgery is common and is associated with severe postoperative pain. The transverse abdominal plane (TAP) block is considered an effective means for pain control in such cases. The quadratus lumborum (QL) block is another option for the management of postoperative pain. The aim of this study was to conduct a meta-analysis and thereby evaluate the efficacy and safety of QL blocks and TAP blocks for pain management after abdominal surgery.

**Methods:**

We comprehensively searched PubMed, EMBASE, EBSCO, the Cochrane Library, Web of Science and CNKI for randomized controlled trials (RCTs) that compared QL blocks and TAP blocks for pain management in patients undergoing abdominal surgery. All of the data were screened and evaluated by two researchers. RevMan5.3 was adopted for the meta-analysis.

**Results:**

A total of 8 RCTs involving 564 patients were included. The meta-analysis showed statistically significant differences between the two groups with respect to postoperative pain scores at 2 h (standardized mean difference [Std.MD] = − 1.76; 95% confidence interval [CI] = − 2.63 to − 0.89; *p* < .001), 4 h (Std.MD = -0.77; 95% CI = -1.36 to − 0.18; *p* = .01),6 h (Std.MD = -1.24; 95% CI = -2.31 to − 0.17; *p* = .02),12 h (Std.MD = -0.70; 95% CI = -1.27 to − 0.13; p = .02) and 24 h (Std.MD = -0.65; 95% CI = -1.29 to − 0.02; *p* = .04); postoperative morphine consumption at 24 h (Std.MD = -1.39; 95% CI = -1.83 to − 0.95; *p* < .001); and duration of postoperative analgesia (Std.MD = 2.30; 95% CI = 1.85 to 2.75; p < .001). There was no statistically significant difference between the two groups with regard to the incidence of postoperative nausea and vomiting (PONV) (RR = 0.55;95% CI = 0.27 to 1.14;*p* = 0.11).

**Conclusion:**

The QL block provides better pain management with less opioid consumption than the TAP block after abdominal surgery. In addition, there are no differences between the TAP block and QL block with respect to PONV.

## Background

There are many kinds of abdominal surgeries, including but not limited to colorectal resection, appendectomy, cesarean section, hysterectomy, and laparoscopic cholecystectomy [1]. Postoperative pain is severe in patients undergoing abdominal surgery, and severe pain not only affects the rate of recovery of patients but also induces a series of pathophysiological reactions [1]. Therefore, it is very important for perioperative patients to have a safe and effective pain management model. Although classic postoperative analgesia methods can provide effective pain relief after surgery, their administration has a well-defined risk of side effects [2–4]. Recently, with the rise in enhanced recovery after surgery, nerve blocks have become the key link in multimodal analgesic regimes [5].

As effective constituents of multimode analgesia, quadratus lumborum (QL) block and transversus abdominis plane (TAP) block are mainly used for postoperative analgesia in abdominal surgery. At present, there have been meta-analyses [6–9] comparing a QL block to a placebo, a TAP block to a placebo, and QL and TAP blocks to other types of analgesia, and the results have shown that TAP blocks and QL blocks can reduce postoperative pain scores, the amount of opioids consumed and opioid-related side effects. Despite the reliability, widespread application and effectiveness of TAP blocks, there are several limitations and complications [10].TAP blocks should not be administered to patients with active infections at the injection site. Other limitations involve the need for a bilateral block for midline incisions and the lack of effectiveness for visceral pain [10].

Compared with TAP blocks, the QL block, which is a regional variation of the TAP block, has been suggested to be a more reliable approach for pain after abdominal surgery. QL blocks result in more extensive sensory blocks than TAP blocks (T10-L3vs.T10-T12, [11]).

Some studies [12–14] have shown that compared with TAP blocks, QL blocks are more effective at postoperative analgesia and can prolong the analgesic time of patients. However, some scholars [15] have confirmed that the analgesic effects of the two treatments are the same in the postoperative period, and there is no difference in the incidence of postoperative adverse reactions. Whether QL blocks offer superior analgesia and faster postoperative recovery than TAP blocks after abdominal surgery remains controversial.

Therefore, the purpose of this study was to evaluate, in the form of a meta-analysis, whether QL blocks or TAP blocks are superior for postoperative pain management and reduce the incidence of adverse reactions after abdominal surgery.

## Methods

The study was a meta-analysis, and ethics approval was not needed. This review and meta-analysis was reported on the basis of the Preferred Reporting Items for Systematic Reviews and Meta-Analyses (PRISMA).

### Search strategy

We searched the following databases (the time limit was from the establishment of the database to September 2019): PubMed, EMBASE, EBSCO, the Cochrane Library, Web of Science and CNKI. We identified randomized controlled trials (RCTs) comparing the use of QL blocks and TAP blocks for analgesia after abdominal surgery. The reference lists within these publications were also investigated to identify other qualified trials not found in the initial database search. No limitations were set with regard to the language of publication. The search terms included “quadratus lumborum block”, “QL block”, “transversus abdominis plane block”, “TAP block”, “abdominal surgery”, “abdominal wall”, “abdominal muscles”, “pain management”, “postoperative pain control”, and “postoperative pain management”.

### Inclusion criteria and study selection

The inclusion criteria were as follow: (1) population: patients undergoing abdominal surgery; (2) study design: RCTs; (3) intervention: QL block; (4) comparison: TAP block; (5) primary outcomes: postoperative pain scores; and (6) secondary outcomes: postoperative opioid consumption, PONV incidence and postoperative analgesia duration. Two reviewers searched for and selected studies according to the abovementioned strategy. The specific process was as follows: (1) retrieved references were deduplicated using Endnote software; (2) screening was initially performed by reading the titles and abstracts; (3) the full texts of the initially identified articles were read, eligible studies were selected and the risk of bias was assessed for each included article; (4) the third researcher made the final decision in any cases of disagreement with respect to the inclusion of studies.

### Data extraction

Two investigators extracted the data from each included study, including basic information (author name, number of cases, sex, age, type of surgery, published year), primary outcomes (pain scores) and secondary outcomes (opioid consumption, postoperative analgesia duration and PONV incidence). All opioids were converted into equianalgesic doses of IV morphine for analysis (IV morphine 10 mg = IVfentanyl 100 μg = IVsufentanil 10 μg) [5]. Pain scores reported as visual, verbal, or numeric rating scale scores were converted to a standardized 0 to 10 analog scale for the quantitative evaluations.

### Assessment of methodological quality

The methodological quality of each RCT was evaluated by two investigators, who used the Cochrane Handbook, and the third researcher made the final decision in any case of disagreement. The following aspects were assessed: random sequence generation, allocation scheme concealment, blinding, accuracy of data results, freedom from selective reporting and other biases. The quality of the outcomes in the meta-analysis was evaluated by the Grading of Recommendations Assessment, Development and Evaluation (GRADE) (Table 1).

**Table 1:** The GRADE evidence quality for main outcomes

Quality assessment							No of patients			
No of Studies	Design	Risk of bias	Inconsistency	Indirectness	Imprecision	Other considerations	QL block groups	TAP block groups	Effect	Quality
Postoperative pain scores at 2 h										
3	RCT	No serious risk of bias	serious	No serious indirectness	No serious imprecision	None	90	90	SMD = −1.76 95%CI: (−2.63 to −0.89)	Moderate
Postoperative pain scores at 4 h										
6	RCT	No serious risk of bias	serious	No serious indirectness	No serious imprecision	None	199	198	SMD = −0.74 95%CI: (−1.34 to − 0.14)	Moderate
Postoperative pain scores at 6 h										
4	RCT	No serious risk of bias	serious	No serious indirectness	No serious imprecision	None	144	143	SMD = −1.24 95%CI: (−2.31 to −0.17)	Moderate
Postoperative pain scores at 12 h										
7	RCT	No serious risk of bias	serious	No serious indirectness	No serious imprecision	None	253	251	SMD = −0.70 95%CI:(−1.27 to − 0.13)	Moderate
Postoperative pain scores at 24 h										
7	RCT	No serious risk of bias	serious	No serious indirectness	No serious imprecision	None	253	251	SMD = −0.60 95%CI: (−1.21 to 0)	Moderate

*GRADE* Grading of Recommendations Assessment, Development, and Evaluation, *RCT* randomized controlled trial, *SMD* standard mean difference, *QL* quadratus lumborum,*TAP* transversus abdominis plane

### Statistical analysis

The statistical analysis was conducted using RevMan 5.3. We performed a heterogeneity test on the included studies and calculated the statistics. When I^2^ was < 0.5 or p was > 0.1, the level of heterogeneity was low, and a fixed-effects model was applied. Otherwise, a random-effects model was used to analyze the sources of heterogeneity. Continuous outcomes are represented as the standardized mean difference (Std.MD) with the associated 95% confidence interval (CI). Dichotomous outcomes are represented as the relative risk (RR) with the associated 95% CI. Due to the limited number (< 10) of included studies, publication bias was not evaluated.

## Results

### Literature search and study characteristics

**Fig. 1:**
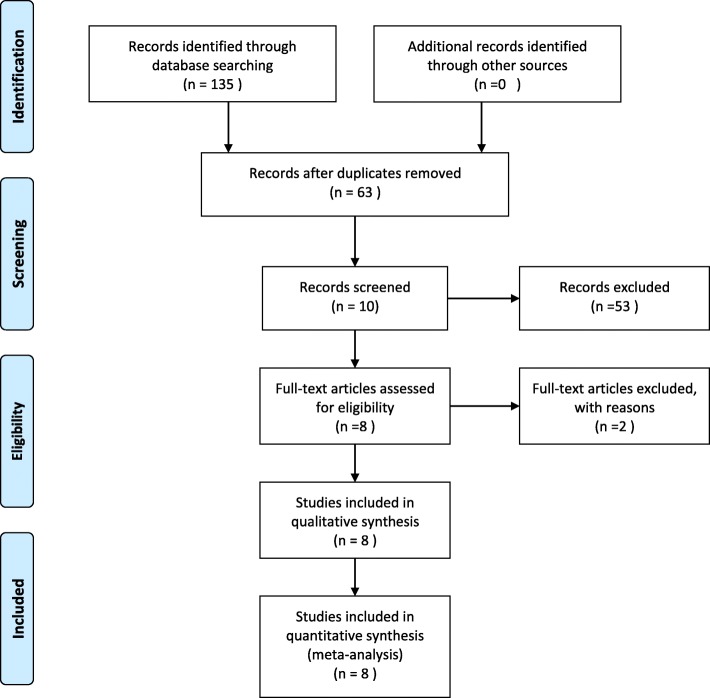
PRISMA Flow Diagram

A total of 135 relevant studies were initially identified, and 8 studies [13–20] were eventually included, with 564 patients. The literature screening process and results are shown in Fig. 1. The basic features of the 8 RCTs included in the meta-analysis are summarized in Table 2.

**Table 2:** Trails characteristics

Author	Research type	Location	Numbers (E/C)	Mean age (E/C)	QL block group	TAP block group	Surgery type	Follow-up
Blanco et al	RCT	UAE	38/38	30.2/31.3	0.125%bupivacaine (0.2 ml/kg)	0.125%bupivacaine (0.2 ml/kg)	Cesarean delivery	4 months
Oksuz et al	RCT	Turkey	25/25	3.13/3.02	0.2% bupivacaine (0.5 ml/kg)	0.2% bupivacaine (0.5 ml/kg)	Low abdominal surgery	5 months
Han et al	RCT	China	39/38	26.3/27.8	20 ml of ropivacaine (concentration of 0.25%)	20 ml of ropivacaine (concentrationof0.25%)	Appendectomy	2 months
Yousef et al	RCT	India	30/30	56.5/50.7	20 ml ofbupivacaine (concentration of 0.25%)	20 ml ofbupivacaine (concentration of 0.25%)	Total abdominal hysterectomy	3 months
Kumar et al	RCT	Egypt	35/35	39.2/38.4	20 ml of ropivacaine (concentration of 0.25%)	20 ml of ropivacaine (concentration of 0.25%)	Low abdominal surgery	2 months
Li et al	RCT	China	40 /40	30/31	20 ml of ropivacaine (concentration of0.375%)	20 ml of ropivacaine (concentration of0.375%)	Cesarean delivery	4 months
Zhu et al	RCT	China	30/30	51/52	20 ml of ropivacaine (concentration of 0.25%)	20 ml of ropivacaine (concentration of 0.25%)	Total abdominal hysterectomy	2 months
Baytar et al	RCT	Turkey	54/53	46.4/48.1	20 ml ofbupivacaine (concentration of 0.25%)	20 ml ofbupivacaine (concentration of 0.25%)	Laparoscopic cholecystectomy	3 months

*E* experimental groups, *C* controlled groups, *RCT* randomized controlled trials, *QL* quadratus lumborum, *TAP* transversus abdominis plane

### Risk of bias

**Fig. 2:**
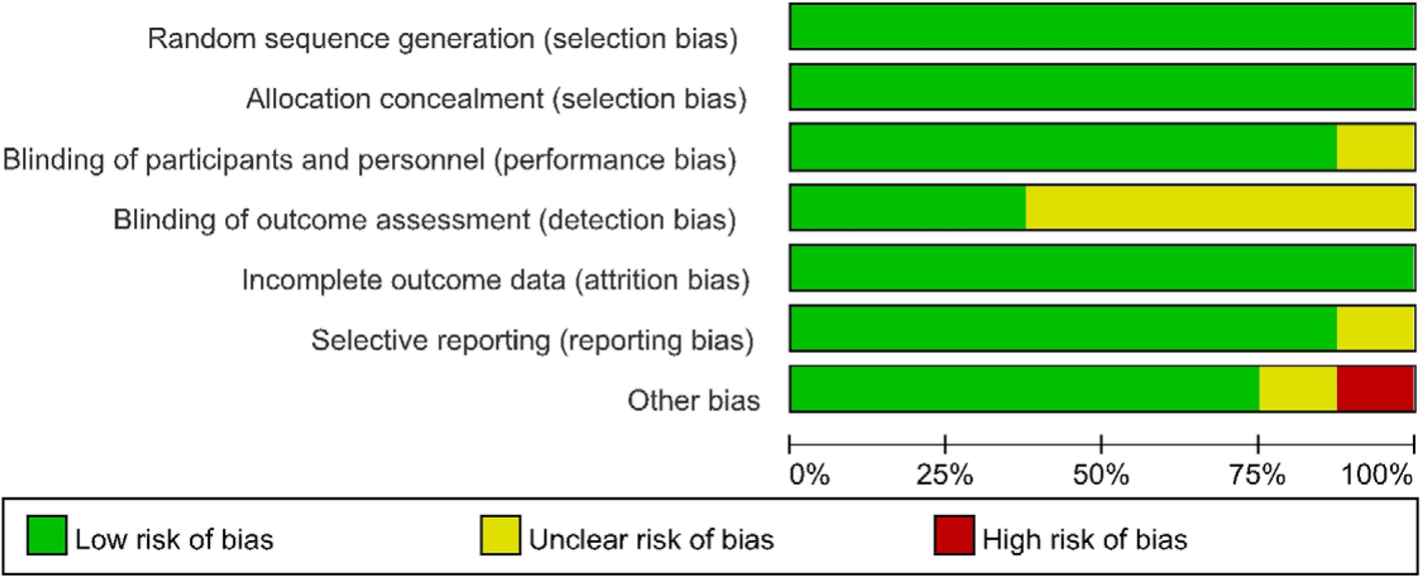
Risk of bias assessment of summary

**Fig. 3:**
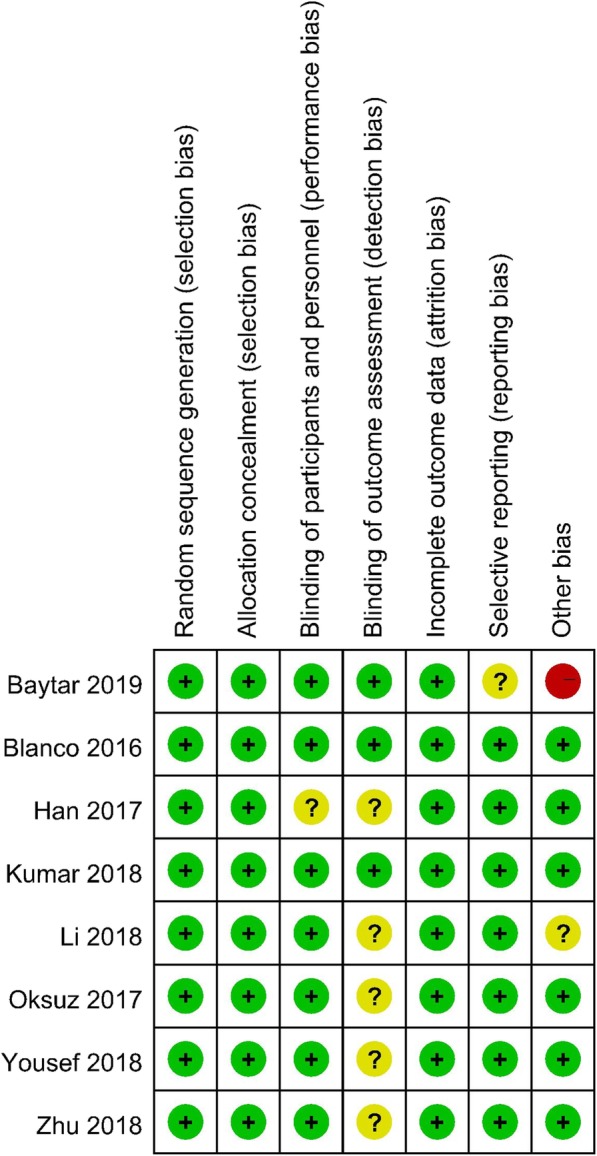
Risk of bias assessment of the studies

The Cochrane Handbook for Systematic Reviews of Interventions was used to evaluate the risk of bias of the RCTs. Five studies [13, 15, 17–19] employed random number tables, two studies [16, 20] adopted computer generated random numbers, and one study [14] used sealed envelopes. All studies described the allocation concealment. One study [19] did not mention the method used to blind the subjects. The researchers were blinded as well. Three studies [16, 18, 20] made use of blinding for outcome measurements, and five studies did not. In addition, all studies reported the completion of the trial without withdrawals. Only one study [20] reported high levels of other biases. (See Fig. 2 and Fig. 3.)

### Outcomes of the meta-analysis

#### Postoperative pain scores at 2 h

**Fig. 4:**
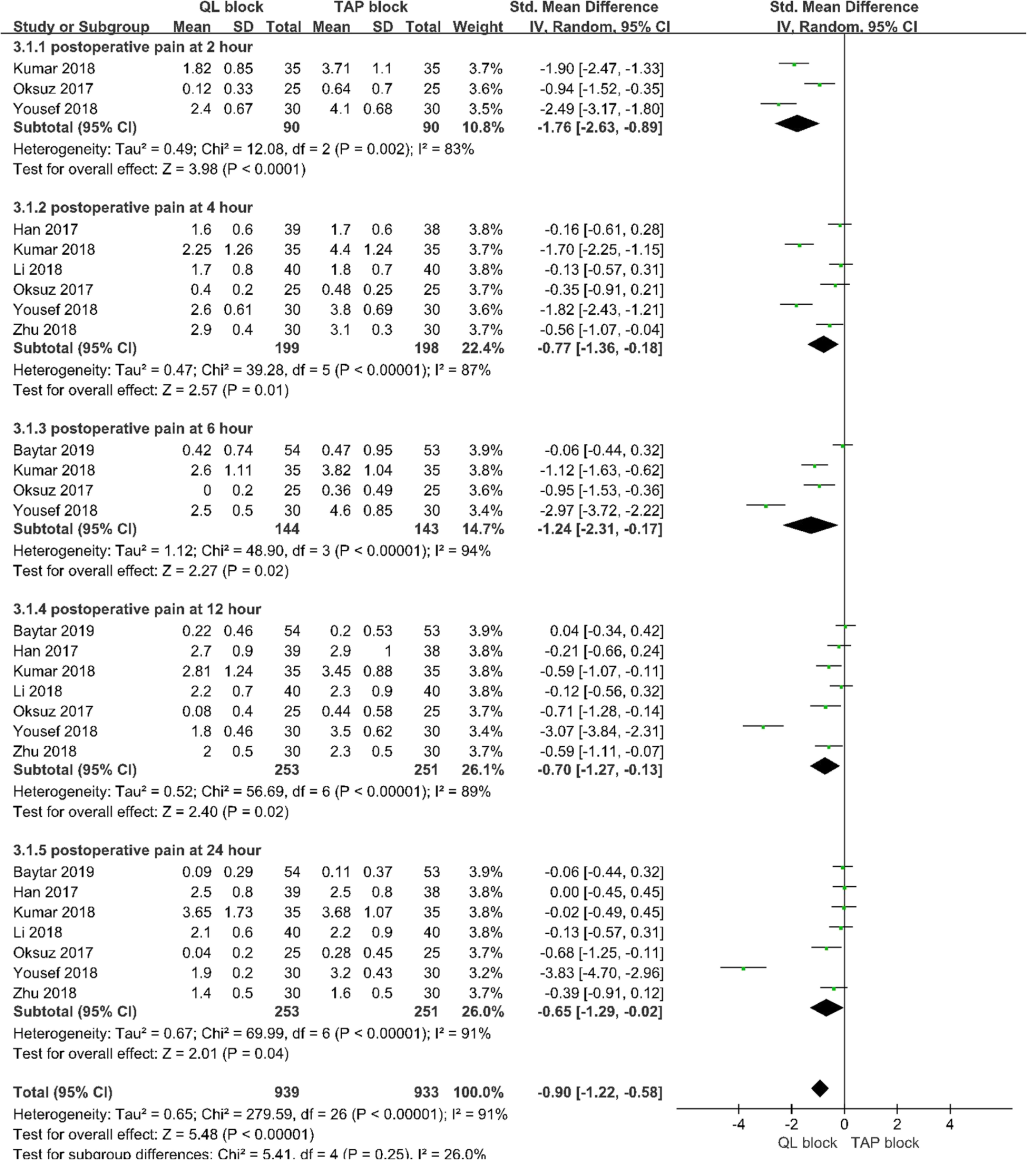
Forest plot for the meta-analysis of postoperative pain scores

Three studies [13, 14, 18] with 180 patients reported pain scores 2 h after abdominal surgery. A random-effects model was used because significant heterogeneity was found among the studies (I^2^ = 0.83, *p* < .10). There was a significant difference in postoperative pain scores at 2 postoperative hours between the 2 groups (Std.MD = -1.76; 95% CI = -2.63 to-0.89; *p* < .001; Fig. 4).

#### Postoperative pain scores at 4 h

Six studies [13–15, 17–19] with 397 patients reported pain scores 4 h after abdominal surgery. A random-effects model was applied because significant heterogeneity was found among the studies (I^2^ = 0.87, *p* < .10). There was a significant difference in postoperative pain scores at 4 postoperative hours between the 2 groups (Std.MD = -0.77; 95% CI = -1.36 to − 0.18; *p* = .01;Fig. 4).

#### Postoperative pain scores at 6 h

Four studies [13, 14, 18, 20] with 287 patients reported pain scores 6 h after abdominal surgery. A random-effects model was applied because significant heterogeneity was found among the studies (I^2^ = 0.94, *p* < .10). There was no significant difference in postoperative pain scores at 6postoperative hours between the 2 groups (Std.MD = -1.24; 95% CI = -2.31 to − 0.17; *p* = .02;Fig. 4).

#### Postoperative pain scores at 12 h

Seven studies [13–15, 17–20] with504 patients reported pain scores 12 h after abdominal surgery. A random-effects model was used because significant heterogeneity was found among the studies (I^2^ = 0.89, *p* < .10). There was a significant difference in postoperative pain scores at 12 postoperative hours between the 2 groups (Std.MD = -0.70; 95% CI = -1.27 to − 0.13; p = .02;Fig. 4).

#### Postoperative pain scores at 24 h

Seven studies [13–15, 17–20] with 504 patients reported pain scores 24 h after abdominal surgery. A random-effects model was adopted because significant heterogeneity was found among the studies (I^2^ = 0.91, *p* < .10). There was a significant difference in postoperative pain scores at 24 postoperative hours between the 2 groups (Std.MD = -0.65; 95% CI = -1.29 to − 0.02; *p* = .04;Fig. 4).

#### Postoperative morphine consumption at 24 h

**Fig. 5:**
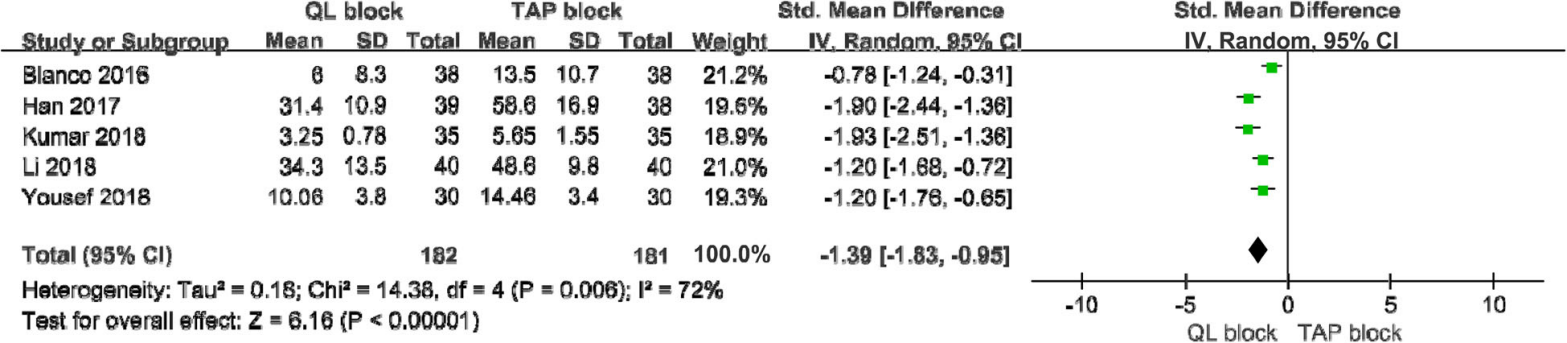
Forest plot for the meta-analysis of postoperative morphine consumption at 24 h

Five studies [13, 15, 16, 18, 19] with 363 patients reported morphine consumption 24 h after abdominal surgery. A random-effects model was used because significant heterogeneity was found among the studies (I^2^ = 0.72, p < .10). There was a significant difference in morphine consumption at 24 postoperative hours between the 2 groups (Std.MD = -1.39;95% CI = -1.83 to − 0.95; *p* < .001; Fig. 5).

### Duration of postoperative analgesia

**Fig. 6:**
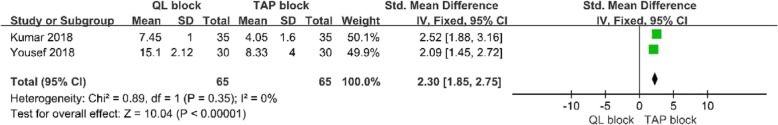
Forest plot for the meta-analysis of duration of postoperative analgesia

Two studies [13, 18] with 130 patients reported the analgesia duration after abdominal surgery. A fixed-effects model was adopted because significant heterogeneity was not found among the studies (I^2^ = 0, *p* > .10). There was a significant difference in postoperative analgesia duration between the 2 groups (Std.MD = 2.30; 95% CI 95% CI = 1.85 to 2.75; p < .001; Fig. 6).

#### Postoperative nausea and vomiting

**Fig. 7:**
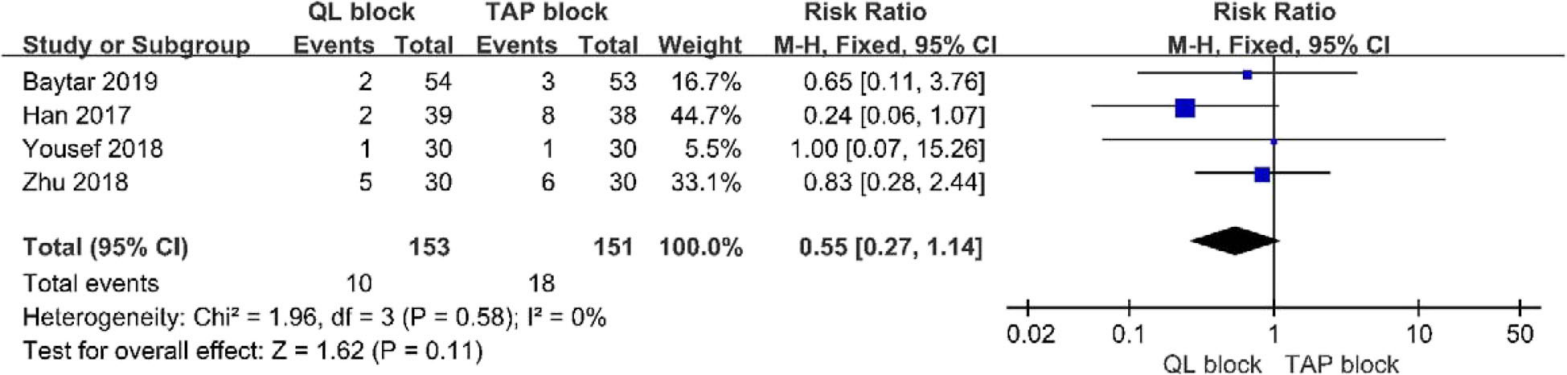
Forest plot for the meta-analysis of PONV

Four studies [16, 17, 19, 20] with304 patients showed the incidence of PONV. A fixed-effects model was used because significant heterogeneity was not found among the studies (I^2^ = 0, p > .10). There was no significant difference in PONV between the 2 groups (RR = 0.55; 95% CI = 0.27 to 1.14; *p* = 0.11;Fig. 7).

## Discussion

The meta-analysis of 8RCTs showed that the pain scores at 2, 4, 6, 12 and 24 postoperative hours were significantly lower in the QL group than in the TAP group. The amount of postoperative morphine consumption was lower in the QL group than in the TAP group. The duration of postoperative analgesia was longer in the QL group than in the TAP group. In addition, there were no differences in PONV.

In the UK, approximately 700,000 people undergo abdominal surgery every year [21]. Patients experience severe pain, which leads to a series of complications. Due to pain and discomfort, patients do not cough and cannot carry out their normal activities, resulting in respiratory complications that may lead to pulmonary infections [4, 22]. If the symptoms are severe, patients may have postoperative delirium, myocardial ischemia and other serious complications. If the pain cannot be controlled in a timely fashion, acute pain can transform into chronic pain, which distresses the patient, affects wound healing, reduces the quality of life of the patient, and prolongs his or her length of hospital stay [23, 24]. Therefore, adequate postoperative analgesia has important clinical significance. In recent years, regional blocks, as a key link in multimodal analgesia, have been increasingly widely used for postoperative analgesia after abdominal surgery. TAP blocks and QL blocks belong to this treatment category [5, 25]. Thus, the potential for effective analgesia after abdominal surgery is becoming increasingly promising.

TAP blocks were first described by Rafi in 2001 [26]. TAP blocks involve the Petit triangle (that is, the lower lumbar triangle: the outer boundary is the posterior edge of the abdominal external oblique muscle, the inner boundary is the leading edge of the latissimus dorsi muscle, and the lower boundary is the iliac crest). The TAP is a nanatomical space between the transverse abdominal muscle and the medial oblique muscle [27]. The thoracolumbar nerve originates from the T6 to L1 segment of the spinal nerve root and innervates the abdominal wall, providing anterolateral sensation. The injection of local anesthetics into this space can block nerve afferents and provide adequate analgesia for the anterolateral abdominal wall [28]. However, due to the narrow range of abdominal transverse muscle plane blocks, they are often limited to use as postoperative analgesia for lower abdominal surgery, and the application of these blocks as postoperative analgesia for upper abdominal surgery is limited. As a new technique for abdominal trunk block, QL blocks were first proposed by Blanco in 2007; anesthetic is injected adjacent to the anterolateral aspect of the QL muscle and its fascia, blocking the posterior abdominal wall [16]. The block level is high (T7-L1), which can provide postoperative analgesia for both upper and lower abdominal surgery. The key to the analgesic effect of a QL block is the thoracolumbar fascia (TLF). The TLF is a complex tubular structure formed by connective tissue. Local anesthetics can spread through the TLF to the paravertebral space to generate an indirect paraspinal block [29, 30]. Therefore, it has an effect on visceral pain and abdominal incision pain. Additional studies [7, 12, 31] have shown that two different trunk blocks have adequate analgesic effects for the management of pain after abdominal surgery. FuscoP [32] et al. confirmed the analgesic effect of TAP blocks after cesarean section. Blanco [16] et al. conducted a RCT of 76 patients after cesarean section to compare the effects of pain management via QL block and TAP block. The results showed that TAP blocks were better able to reduce postoperative morphine requirements. However, there was no significant difference in postoperative pain scores between the two groups. In addition to clinical trials, other meta-analyses have confirmed the feasibility of the use of TAP blocks and QL blocks as analgesia after abdominal surgery.

Previous studies have reported the effectiveness and safety of QL blocks and TAP blocks for postoperative pain management after abdominal surgery. However, it is not yet clear which option is better. Zhu [17] et al. found no significant difference in VAS scores between patients receiving QL blocks and those receiving TAP blocks 4 h and 8 h after surgery, while the resting and motor scores 12 h and 24 h after surgery were lower in the QL block group than in the TAP block group. However, Oksuz [14] et al. reported that QL blocks provided superior analgesic relief. They compared the numbers of patients who needed analgesia in the first 24 h and the pain scores at 30 min and 1, 2, 4, 6, 12, and 24 h(s), and they found that the QL block was significantly superior to the TAP block. At the same time, Kumar’s study [18] demonstrated that the pain scores of the patients in the QL block group were lower than those of the patients in the TAP block group 2, 4, 8, 12 and 24 h after lower abdominal surgeries.

In contrast to the above studies, we systematically evaluated the analgesic effects and adverse reactions of QL blocks and TAP blocks to determine which is the better regional blocking technique for pain management after abdominal surgery. The results of our meta-analysis, which included 8 RCTs, indicated that the QL block is superior to the TAP block with respect to the analgesic effect at 2, 4, 6, 12 and 24 h after surgery. Overall, the present study suggests that the effect of the QL block is better than that of the TAP block for the early management of pain after abdominal surgery. We found that the QL block is superior to the TAP block with regard to reducing postoperative opioid requirements and that pain control lasts longer after the QL block, which is consistent with the findings of Blanco et al. The reason may be that the TLF is formed by the arrangement of the anterior, middle and posterior layers. After the posterior layer and the middle layer meet at the lateral edge of the vertical spinal muscle, they converge with the anterior layer at the lateral edge of the lumbar quadratus muscle to form the aponeuros is starting point of the transverse abdomen muscle. When QL block is performed, the local anesthetics can spread not only within the TLF but also to the abdominal transverse muscle plane and paraspinal space, creating an effect similar to the effect of a paravertebral nerve block [33]. The TLF has receptors that can regulate autonomic nerve function and pain mechanisms. Local anesthetics applied to the QL block some sympathetic nerves and thereby achieve a better effect. There was no significant difference in the incidence of PONV between the two groups. The reasons may be related to the different methods of anesthesia but may also stem from the sample size; therefore, a large number of consistent clinical trials are still needed.

Regarding the sensitivity analysis, there was still significant heterogeneity when performing the analysis by omitting one study in turn and when performing subgroup analyses. The main reasons for heterogeneity include the following: (1) Five RCTs originated in Asia, and the patient sample of one of the RCTs was limited to children. There may be relevant differences in the analytical results of the integrated data.(2) The types of surgery varied, including cesarean sections, total abdominal hysterectomies and appendectomies. The degree of postoperative pain varies among patients undergoing different abdominal surgeries. (3) The anesthetic drugs and concentrations used in the RCTs were different. Bupivacaine was used in 4 RCTs at concentrations of 0.125, 0.2 and 0.25%. The concentrations of ropivacaine used in the other 4 RCTs were 0.25 and 0.375%. (4) Three RCTs used subarachnoid anesthesia, and five RCTs employed general anesthesia.

The limitations of this meta-analysis are as follows: in the data extraction, some observation indexes in the literature were only reported as the mean and median or in the form of graphics and text; thus, these results could not be included in the analysis, which may have excluded some high-quality studies. Furthermore, there was no explicit mention of the optimal drug type and concentration for the two trunk plane blocks, which need to be further studied to arrive at a satisfactory approach. During the data collection process, the original data from requested from the author by e-mail, but no response was received. Finally, although our meta-analysis has shown that there is a statistically significant difference in postoperative pain scores between patients receiving QL blocks and TAP blocks, a difference in pain scores that is less than 2 points has limited clinical relevance. Further studies are needed to clarify the more subtle clinical differences in pain after receiving a QL block compared with a TAP block after abdominal surgery.

## Conclusions

Compared with the TAP block, the QL block provides better pain management with less opioid consumption after abdominal surgery. However, further large RCTs are needed to confirm these findings.

## Data Availability

The datasets used and/or analyzed in the current study are availablefrom the corresponding author on reasonable request.

## Abbreviations

CI: Confidence interval, St.
MD: Standard mean difference
PONV: Postoperative nausea and vomiting
RCT: Randomized controlled trial
RR: Relative risk
VAS: visual analogue scale
